# Prognostic value of CD8CD45RO tumor infiltrating lymphocytes in patients with extrahepatic cholangiocarcinoma

**DOI:** 10.18632/oncotarget.25163

**Published:** 2018-05-04

**Authors:** Richard Kim, Domenico Coppola, Emilie Wang, Young Doo Chang, Yuhree Kim, Daniel Anaya, Dae Won Kim

**Affiliations:** ^1^ Department of Gastrointestinal Oncology, Moffitt Cancer Center, Tampa, FL, USA; ^2^ Department of Anatomic Pathology, Moffitt Cancer Center, Tampa, FL, USA; ^3^ Department of Internal Medicine, University of South Florida, Tampa, FL, USA; ^4^ Department of Supportive Care, Moffitt Cancer Center, Tampa, FL, USA; ^5^ Department of Population Medicine, Harvard Medical School, Boston, MA, USA

**Keywords:** cholangiocarcinoma, tumor infiltrating lymphocytes, tumor microenvironment, PD-L1

## Abstract

Cholangiocarcinoma is a malignancy arising from the biliary tract epithelial cells with poor prognosis. Tumor infiltrating lymphocytes (TIL)s and programmed cell death receptor ligand 1 (PD-L1) have a prognostic impact in various solid tumors. We aimed to investigate TILs and PD-L1 expression and their clinical relevance in cholangiocarcinoma. Tumor samples from 44 patients with resected and histologically verified extrahepatic cholangiocarcinoma were evaluated for CD8, CD45RO and PD-L1 expression, and their correlations with clinicopathological data and survival data were analyzed. Total 44 extrahepatic cholangiocarcinoma tissues were evaluated. CD8+ tumor infiltrating lymphocytes (TIL)s were observed in 30 (68%) tumors. Among them, 14 had CD8+CD45RO+ TILs. PD-L1 was expressed on cancer cells in 10 (22.7%) tumors in 34 evaluable extrahepatic cholangiocarciniomas. The presence of CD8+ TILs or CD8+CD45RO+ TILs was not associated with clinical staging or tumor differentiation. Extrahepatic cholangiocarcinoma with CD8+CD45RO+ TILs had longer overall survival (OS) on univariate (*P* = 0.013) and multivariate (*P* = 0.012) analysis. Neither CD8+TIL nor PD-L1 expression on cancer cells correlated significantly with OS. These results add to the understanding of the clinical features associated with CD8 TILs and PD-L1 expression in extrahepatic cholangiocarcinoma, and they support the potential rationale of using PD-1 blockade immunotherapy in cholangiocarcinoma.

## INTRODUCTION

Cholangiocarcinoma is a rare cancer originating from the epithelial cells of the intrahepatic and extrahepatic bile ducts. Extrahepatic cholangiocarcinoma is further characterized as perihilar and distal cholangiocarcinoma. Cholangiocarcinoma is an uncommon malignancy accounting for approximately 3% of all gastrointestinal tumors [1]. However, it is a very aggressive group of malignancies with overall dismal prognosis. The 5 year survival rate is only 10% [2]. While local treatment such as surgical resection is effective for localized disease, treatment of recurrent or metastatic disease is quite challenging. Although gemcitabine plus cisplatin has demonstrated a significant antitumor activity as first-line therapy for metastatic cholangiocarcinoma [3], new effective therapeutic approaches are needed for further improvement of clinical outcome.

Rapid advances in tumor immunology have improved our understanding of key regulators that mediate antitumor T cell responses, leading to the development of new immunotherapeutic approaches targeting programmed cell death 1 (PD-1). PD-1 is one of the negative immune regulators which play an essential role in immunosuppression of antitumor immunity in local tumor environment. PD-1 is expressed on the surface of activated T cells, and its ligand, PD-L1 is broadly displayed on antigen presenting cells and tumor cells [4]. The ligation of PD-1 and PD-L1 inhibits T cell proliferation and activation and induces apoptosis of tumor antigen-specific T cells to inhibit antitumor immunity and escape immune surveillance [4]. Emerging clinical data have demonstrated durable clinical activity and safety of PD-1 blockade agents in diverse malignancies including melanoma, non-small cell lung cancer, renal cell carcinoma, urothelial cancer, Hodgkin lymphoma, head and neck cancer and mismatch repair deficient colorectal cancer [5]. The remarkable success of PD-1 blockade immunotherapy has led to the extensive research effort to discover prognostic and predictive markers and to evaluate potential therapeutic activity of antiPD-1 immunotherapy in other malignancies. Recent clinical data have demonstrated that presence of CD8 tumor infiltrating lymphocytes (TILs) and PD-L1 expression in tumor microenvironment have not only prognostic implication but also predict clinical response to PD-1 blockade immunotherapy in a number of malignant tumors [6, 7]. However, very little is known about the immune microenvironment of cholangiocarcinoma.

We have performed a retrospective analysis of a single institution cohort of extrahepatic cholangiocarcinoma patients with expression of TILs and PD-L1 in tumor microenvironment to improve our understanding of their significance and to provide potential rationale for the utility of PD-1 blockade immunotherapy option in cholangiocarcinoma.

## RESULTS

### Baseline characteristics

A total of 44 patients who underwent surgical resection of extrahepatic cholangiocarcinoma were identified (Table 1). The median age of the patients was 67 years (range 42–86) and 52.3% were male. Eleven (25%) patients had perihilar and 33 (75%) had distal tumor. A majority of tumors (84.0%) were well to moderately differentiated and R0 resection was done on 37 (84.1%) patients.

**Table 1:** Patients characteristics

Variable	*N* = 44
**Median Age (range)**	67 (42–86)
**Gender**	
Male (%)	23 (52.3%)
Female (%)	21 (47.7%)
**Location**	
Perihilar (%)	11 (25%)
Distal (%)	33 (75%)
**Stage**	
I (%)	12 (27.3%)
II (%)	25 (56.8%)
III (%)	7 (15.9%)
**Differentiation**	
Well (%)	13 (29.5%)
Moderate (%)	24 (54.5%)
Poorly (%)	7 (15.9%)
**Surgical Resection**	
R0 (%)	37 (84.1%)
R1 (%)	5 (11.4%)
R2 (%)	2 (4.5%)

### TIL and PD-L1 expression in tumor microenvironment

**Figure 1:**
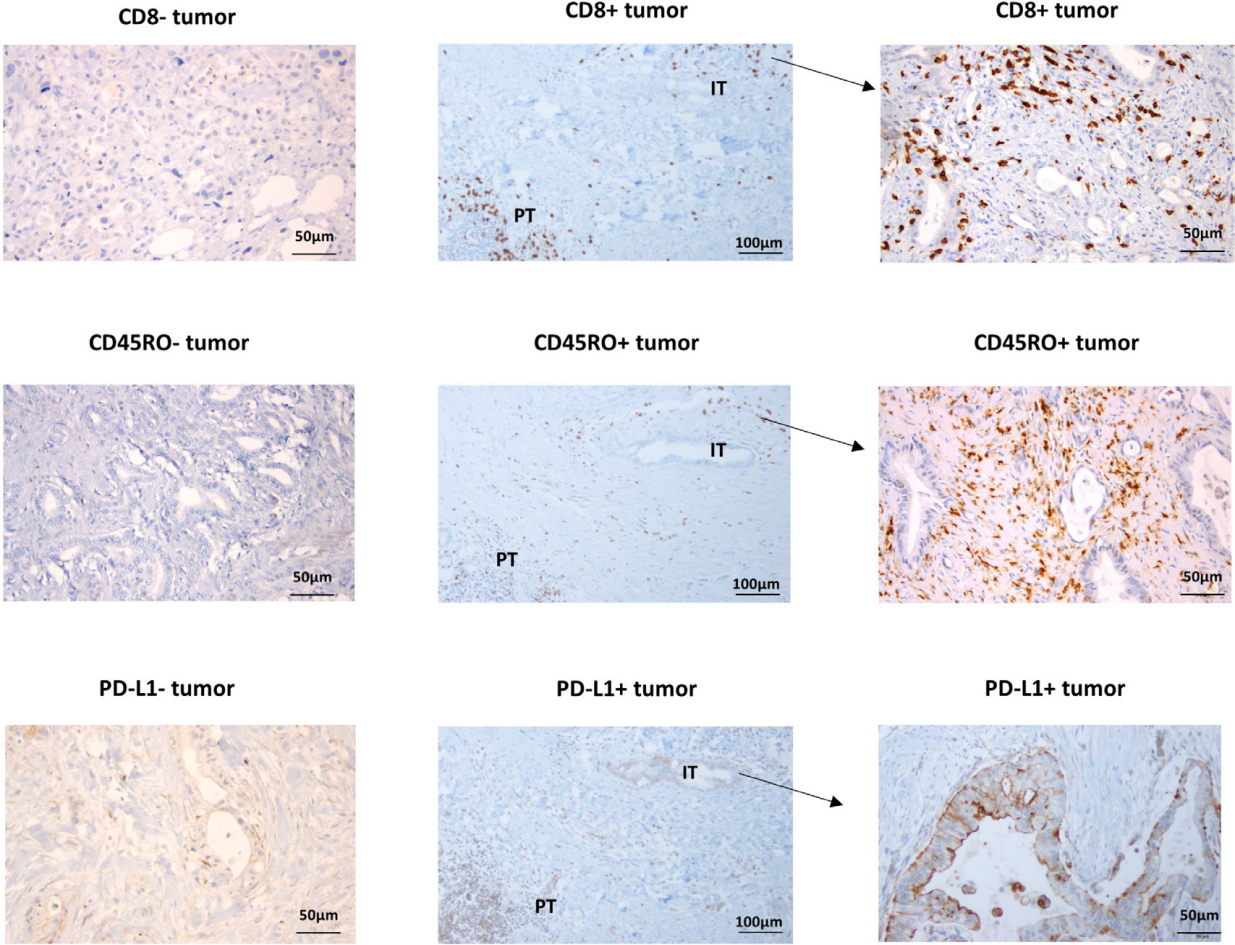
Representative immunohistochemical staining of CD8, CD45RO and PD-L1 IT: intratumoral area, PT: peritumoral area.

Tumor microenvironment was evaluated using immunohistochemical staining with anti-CD8 and PD-L1 antibodies. PD-L1 expressing tumor and immune cells and CD8+ lymphocytes were identified in intratumoral and peritumoral area (Figure 1). Intratumoral CD8+ TILs were observed in 30 (68.2%) tumor samples. While 10 (90.9%) out of 11 perihilar tumors have intratumoral CD8+TIL, 20 (60.6%) out of 33 distal tumors have it. PD-L1 expression was evaluated only in 34 tumors (perihilar: 9, distal: 25) due to lack of sufficient tumor tissues in the remaining samples. While PD-L1 was expressed in 32 (94.1%) tumor samples, PD-L1 expression on cancer cells was observed in only 10 tumors (22.7%). All 10 tumors with PD-L1 expressing cancer cells had CD8+ TIL infiltration. Two of them are perihilar and the rest are distal disease. Previous reports suggested CD45RO expressing immune cell infiltration as an independent prognostic marker in gastrointestinal cancer including colorectal and gastric cancer [8, 9]. Tumor infiltrating CD45RO+ immune cells were also evaluated, and they were detected in 21 (47.7%) of cholangiocarcinomas. Fourteen tumors have CD8+CD45RO+ T cells (memory CD8 T cells) in 21 extrahepatic tumors with CD45RO+ infiltrating immune cells.

### Clinical and pathologic characteristics associated with tumor-immune cell infiltration

The relationship between clinicopathological features and immune cell infiltration was analyzed (Supplementary Figure 1). The presence of CD8+ TILs or CD8+CD45RO+ TILs was not associated with clinical staging or tumor differentiation in extrahepatic cholangiocarcinoma. No correlation between PD-L1 expression and clinical staging or tumor differentiation was observed (data not shown).

### Prognostic value of tumor-immune cell infiltration and PD-L1 expression

**Figure 2:**
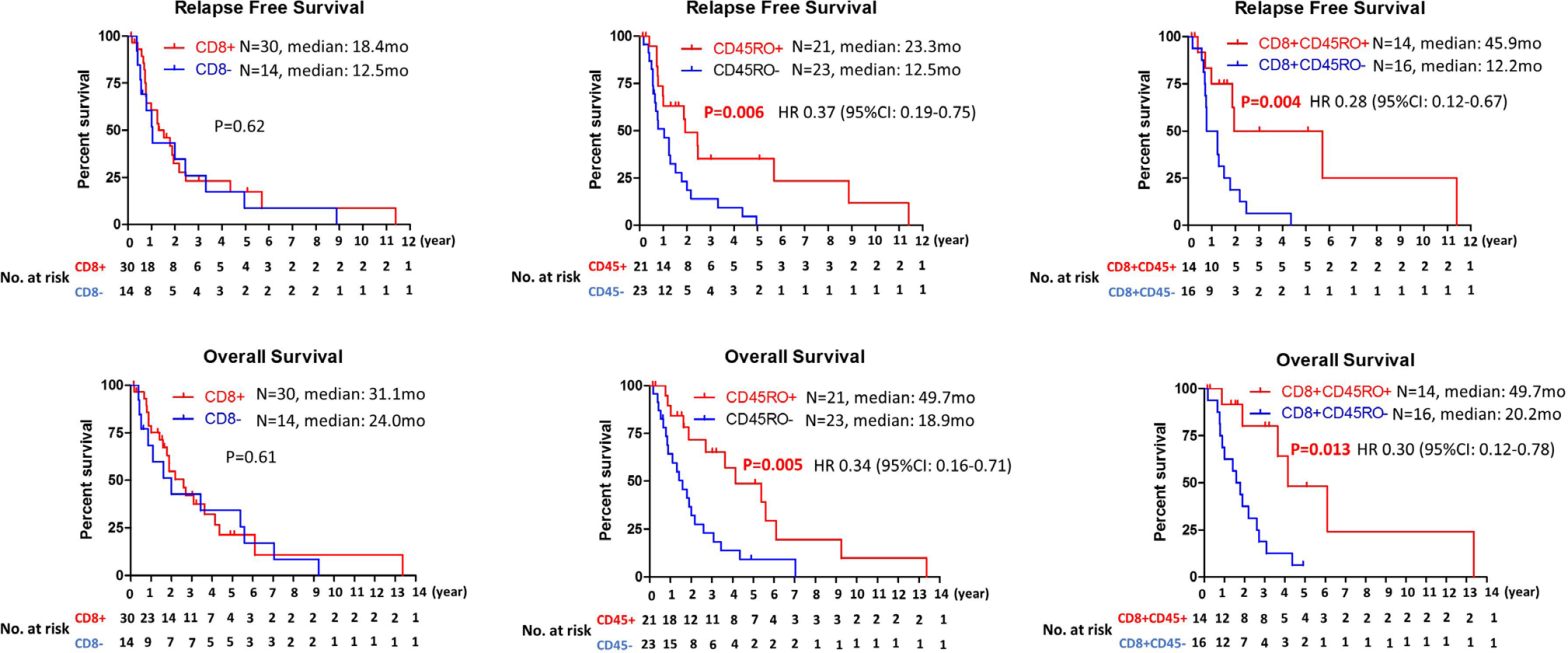
Kaplan–Meier analysis of relapse free survival and overall survival based on the presence of CD8, CD45RO or CD8CD45RO tumor infiltrating lymphocytes

**Figure 3:**
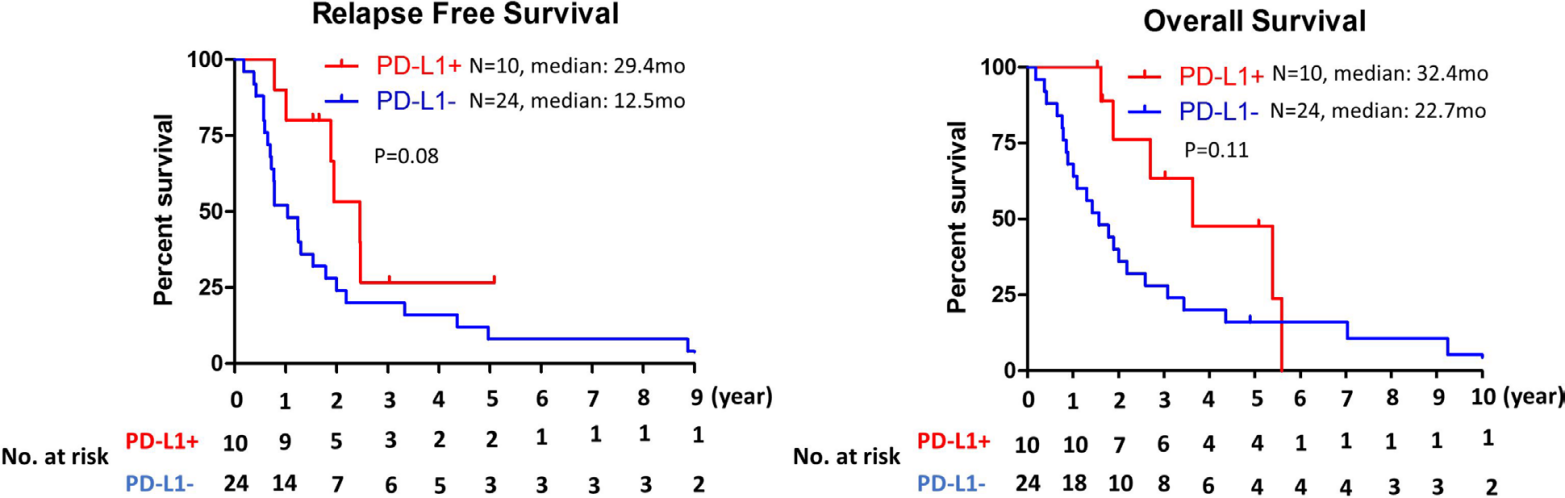
Kaplan–Meier analysis of relapse free survival and overall survival by PD-L1 expression status on tumor cells

To determine prognostic value of immune cells infiltration, correlation between immune cells infiltration and clinical outcome was examined. While no significant correlation was observed between CD8+ TILs and relapse free survival (RFS) or overall survival (OS), patients with CD45RO+ infiltrating immune cells had longer relapse free survival and overall survival in cholangiocarcinoma (median RFS: 23.3 mo vs 12.5 mo, *P* = 0.006; median OS: 49.7 mo vs 18.9mo, *P* = 0.003) (Figure 2). To further evaluate clinical outcomes of tumors with CD8+ TILs, CD8+ TILs were further subgrouped into CD8+ TILs high (≥100 positive cells per high power field) and low (<100 positive cells per high power field) or CD8+CD45RO+ TILs and CD8+CD45RO– TILs. While high number of CD8+ TILs infiltration did not have any prognostic value (Supplementary Figure 2), significant correlation between the presence of CD8+CD45RO+ TIL and RFS or OS (median RFS: 45.9 mo vs 12.2 mo, *P* = 0.004; median OS: 49.7 mo vs 20.2 mo, *P* = 0.019) was observed (Figure 2). PD-L1 expression on cancer cells was not associated with RFS or OS (Figure 3). For evaluation of the relationship between the level of PD-L1 expression and clinical outcome, the PD-L1 expressing tumors were divided into two groups (high vs low) using cutoff value of 5%. No correlation between the level of PD-L1 expression and RFS or OS was observed (Supplementary Figure 3).

### Univariate and multivariate analysis

Univariate and multivariate Cox regression modeling was performed to identify significant predictors of OS including age, gender, resection, differentiation, clinical stage, CD8+ TILs, CD8+CD45RO+ TILs and CD45RO+ infiltrating immune cells (Table 2). Resection, stage, CD8+CD45RO+ TILs and CD45RO+ infiltrating immune cells were associated with survival on univariate analysis. On multivariate analysis, the presence of CD8+CD45RO+ TILs (HR 0.29, 95% CI 0.11–0.76, *P* = 0.012) as well as resection and stage were independent prognostic markers. While the presence of CD45RO+ infiltrating immune cells was associated with improved survival on univariate analysis, it was not an independent prognostic marker on multivariate analysis.

**Table 2:** Univariate and multivariate analysis using Cox Proportional Hazards Model

Variables	Univariate			Multivariate		
	HR	95% CI	*P* value	HR	95% CI	*P* value
**Age**	1.01	0.97–1.04	0.67			
**Gender**						
Male						
Female	0.81	0.39–1.70	0.58			
**Resection**						
R0						
R1	0.37	0.09–1.57	0.18			
R2	4.9	1.09–21.97	0.038	7.66	1.45–40.59	0.017
**Differentiation**						
Well						
Moderately	1.64	0.73–3.67	0.23			
Poorly	3.8	0.88–16.46	0.08			
**Stage**						
I						
II	1.9	0.80–4.47	0.14			
III	3.54	1.19–10.56	0.023	4.73	1.49–14.99	0.008
**CD8**	0.83	0.41–1.70	0.61			
**CD8CD45RO**	0.3	0.12–0.78	0.013	0.29	0.11–0.76	0.012
**CD45RO**	0.34	0.16–0.71	0.005	0.65	0.24–1.75	0.39

Abbreviation: CI: confidence interval.

## DISCUSSION

The tumor immune microenvironment is a potentially powerful predictive and prognostic marker of immunotherapy. The recent success of PD-1 blockade immunotherapy has led to extensive studies to discover predictive markers in tumor microenvironment of several malignancies. However, only limited data of tumor immune microenvironment are available in cholangiocarcinoma. While the presence of TILs and PD-L1 expression have been identified in cholangiocarcinoma [10, 11], their clinical associations have not been fully elucidated due to the rarity of the disease. In this study, we evaluated tumor immune microenvironment of 44 resected extrahepatic cholangiocarcinoma tumor samples with the clinical outcome. Our study focused on CD8 TILs and PD-L1 expression as they can provide potential rationale for the usage of PD-1 blockade immunotherapy.

In our study, intratumoral CD8 TILs were observed in 68% of extrahepatic cholangiocarcinomas which are similar to the frequency observed in the previous report [10]. Interestingly, only CD8+CD45RO+ TIL but not CD8+ TIL had prognostic significance. The presence of memory (CD45RO+) CD8 TILs has been reported as a favorable prognostic marker in several cancers [12]. Our results did not demonstrate prognostic value of CD8 TILs. This was in contrast with other cancers where improved survival was associated with CD8 TILs [13]. One possible explanation for the discrepancy is the presence of immune suppressive cells such as regulatory T cells (Treg)s and suppressor CD8 T cells. Previous meta-analysis data showed CD8/Tregs (FOXP3+) ratio produced more impressive hazard ratio than CD8 TILs alone [13], and Tregs but not CD8 TILs were reported as independent prognostic markers in cholangiocarcinoma [14]. In addition, recent data demonstrated a majority of CD8 suppressor cells do not express CD45RO [15], which may explain our result that memory type CD8 TIL (CD8+CD45RO+) but not CD8 TIL was associated with significant improvement in OS. Unfortunately, we could not examine FOXP3 due to insufficient tumor samples.

PD-L1 expression was observed on both immune cells and cancer cells. Total 22.7% of tumors had PD-L1 expression on their cancer cells which is relatively similar to the rate observed in a previous report (29.6%) [11]. However, it is higher than other cholangiocarcinoma studies (9% and 11.6%) [10, 16]. The discordance of PD-L1 expression may be explained by different anti-PD-L1 antibodies and different cut-off value of PD-L1 staining positivity in each study. In addition, different cholangiocarcinoma patient population in each study may contribute to the discrepancy since PD-L1 is highly expressed on special subtypes of cholangiocarcinomas such as intrahepatic lymphoepithelioma like cholangiocarcinoma [17], occupational cholangiocarcinoma [18] and liver fluke related cholangiocarcinoma [19] compared with conventional cholangiocarcinoma.

We did not observe any correlation between PD-L1 expression on cancer cells and overall survival which is consistent with previous reports [11, 16, 19]. However, other studies suggested PD-L1 expression as a negative prognostic factor in cholangiocarcinoma [20, 21]. This discrepancy may be explained by small sample size of each study ranging from 27–70 cases. In addition, they were all retrospective studies conducted mainly from single institutions.

In our study, we combined perihilar and distal tumors to extrahepatic cholangiocarcinoma due to small sample size, which did not allow direct comparison of each subtype of cholangiocarcinoma.

In conclusion, our study reports the analysis of tumor microenvironment in patients diagnosed with extrahepatic cholangiocarcinoma. Our results suggest that CD8 memory (CD8+CD45RO+) TILs but not CD8 TILs correlate with a favorable prognosis in extrahepatic cholangiocarcinoma. In addition, our study show that cholangiocarcinoma cancer cells express PD-L1 which is suggested as a potential predictive marker of PD-1 blockade immunotherapy. It is unclear if any of these markers has predictive value on the response to immunotherapy but hopefully ongoing immunotherapy trials will shed some light on these questions.

## MATERIALS AND METHODS

### Patient selection and clinical data collection

Under an Institutional Review Board-approved protocol, formalin-fixed paraffin-embedded tumor samples from patients with resected and histologically verified extrahepatic cholangiocarcinoma at Moffitt Cancer Center, between 1990 and 2015 were obtained. Patient demographics, primary tumor characteristics, treatment received and overall survival were collected by chart review retrospectively.

### Immunohistochemical staining

Tissue sections (4μm thick) were immersed in citrate buffer solution for antigen retrieval and boiled in microwave for 10 min and washed in buffer solution. They were incubated with primary antibody including anti-CD8 (C8/144B, DAKO), anti-CD45RO (UCHL1, DAKO) and the anti-PDL1 mouse IgG1 (clone 5H1; Thompson) antibodies for 1 hr at room temperature and then washed in buffer solution. After 1 hr of incubation in the secondary antibody, the sections were incubated with streptavidin-biotin-complex (DAKO). Appropriate positive and negative controls were used. Stained tumor samples were reviewed and enumerated by a GI pathologist. For estimation of positive reaction, only the strongly stained nuclei were counted as a percentage of all tumor nuclei. Less than 1% were estimated as negative. Samples with greater than 1% of cells exhibiting strong nuclear staining were considered positive.

### Statistical methods

Fisher's exact test was used to assess the association between TILs and clinicopathological characteristics. The association between expression of CD8/CD45RO or PD-L1 and survival was investigated using Kaplan-Meier survival, and Log-rank testing was performed to determine the significance of observed difference. COX proportional hazard regression was used for multivariate analysis. All statistical analyses were performed using IBM SPSS Statistics 24. All statistical tests used a significance level of 5%.

## SUPPLEMENTARY MATERIALS AND FIGURES



## Abbreviations

OS: overall survival
PD-1: programmed cell death receptor
PD-L1: programmed cell death receptor ligand 1
RFS: relapse free survival
TIL: tumor infiltrating lymphocyte
Treg: regulatory T cell
