# Supplementary material for: Prognostic value of CD8CD45RO tumor infiltrating lymphocytes in patients with extrahepatic cholangiocarcinoma
Source: Oncotarget. 2018 May 4;9(34):23366–72. doi: 10.18632/oncotarget.25163 (PMC5955104; doi:10.18632/oncotarget.25163)
Supplement: Supplementary file 1 [file oncotarget-09-23366-s001.pdf]

Prognostic value of CD8CD45RO tumor infiltrating lymphocytes in patients with extrahepatic cholangiocarcinoma

SUPPLEMENTARY MATERIALS

	N=44					
	CD8-	CD8+	P value	CD8+CD45RO-	CD8+CD45RO+	P value
Stage			0.55			0.52
I	4	8		3	5	
II	9	16		9	7	
III	1	6		2	4	
Differentiation			0.31			0.6
Well	2	11		6	5	
Moderate	9	15		7	8	
Poorly	3	4		3	1	

Supplementary Figure 1: Relationship between clinicopathological features and immune cell infiltration.

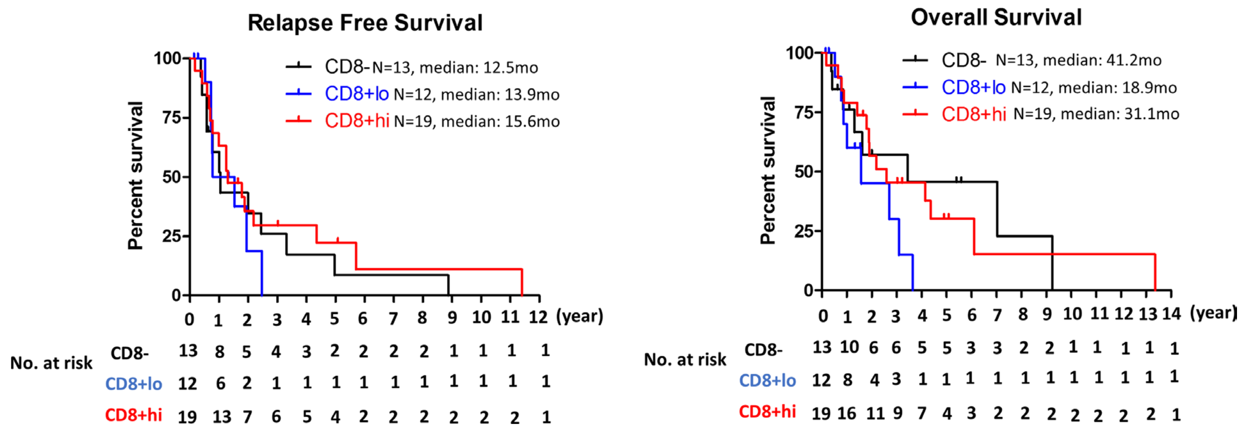

Supplementary Figure 2: Correlation between the presence of CD8+CD45RO+ TIL and RFS or OS.

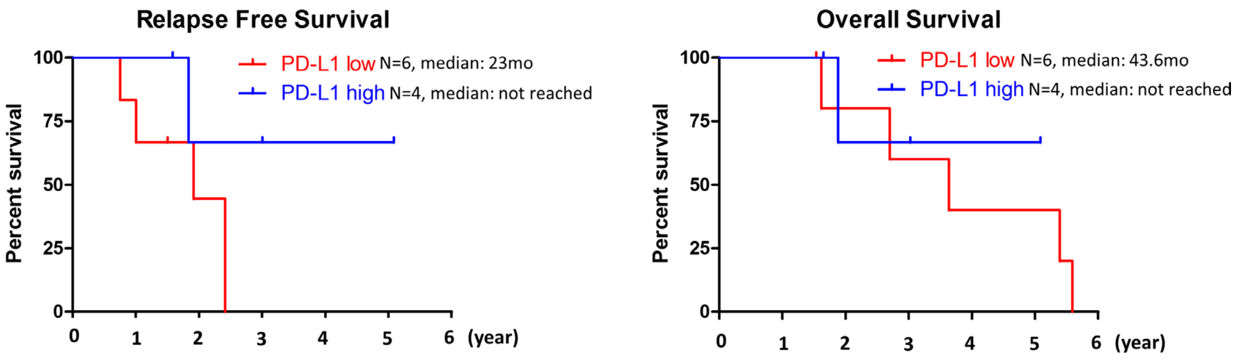

Supplementary Figure 3: Correlation between the level of PD-L1 expression and RFS or OS.
